# Effects of plasma-activated water on germination ‎and initial seedling growth of wheat

**DOI:** 10.1371/journal.pone.0312008

**Published:** 2025-01-24

**Authors:** Ahad Asghari, Elnaz Sabbaghtazeh, Nasrin Roshan Milani, Mohammad Kouhi, Alireza Ahangarzadeh Maralani, Parvin Gharbani, Alireza Sotoudeh Khiaban

**Affiliations:** 1 Department of Physics, Tabriz Branch, Islamic Azad University, Tabriz, Iran; 2 Industrial Nanotechnology Research Center, Tabriz Branch, Islamic Azad University, Tabriz, Iran; 3 Department of Soil Science, Tabriz Branch, Islamic Azad University, Tabriz, Iran; 4 Sustainable Development Management Research Center of Urmia Lake Basin and Aras River, Tabriz Branch, Islamic Azad University, Tabriz, Iran; 5 Department of Physics, National University of Skills (NUS), Tehran, Iran; 6 Department of Chemistry, Ahar Branch, Islamic Azad University, Ahar, Iran; 7 Biophotonic Research Center, Tabriz Branch, Islamic Azad University, Tabriz, Iran; Universidad Autónoma Agraria Antonio Narro, MEXICO

## Abstract

In this research, the effect of seed halopriming with plasma activated water (PAW) on wheat germination parameters have been studied. Response surface methodology was used to investigate the effect of three factors including: 1) type of water (distilled water, 0.2 and 0.4 min/mL PAW), 2) priming time (1, 3 and 5 h), and 3) salinity of the priming solution (0, 25 and 50 mmol/L NaCl) on wheat germination parameters. The results revealed that increasing PAW levels up to 0.18 min/mL led to an increase in seed germination percentage, seedling weight (both fresh and dry), seedling length, vigor indexes A and B, and water uptake and decreasing mean germination time. Increasing the level of PAW by more than 0.18 min/mL had a negative effect on these parameters. The fresh and dry weight of the seedlings respectively at the 0.18 and 0.2 min/mL levels of PAW, in all salinity levels, had the highest value. The effect of halopriming on enhancing seedling length was more than other characteristics. The optimum ranges of PAW, salinity and priming time were 0.13 min/mL, 10.3 mmol/L and 120.22 min for germination percentage, 0.18 min/mL, 15 mmol/L and 191.24 min for seedling fresh weight, 0.15 min/mL, 13.84 mmol/L and 221.2 min for seedling dry weight, 0.33 min/mL, 24.36 mmol/L and 152.62 min for mean germination time, 0.11 min/mL, 19.03 mmol/L and 177.77 min for vigor indexes A and 0.1 min/mL, 18.1 mmol/L and 178.99 min for vigor indexes B.

## Introduction

It is estimated that by the year 2050, the population on Earth will reach nearly ten billion people [1]. ‎Therefore, the global demand for food consumption and healthy agriculture will increase. On the ‎other hand, according to the reports of the Food and Agriculture Organization of the United ‎Nations, food shortages will increase due to the following reasons: climate change, urbanization, ‎industrialization, severe reduction of suitable agricultural land, and an ‎increase of micropollutants, pesticides, and chemicals in the world [2]. It is believed that increasing the rate of ‎germination and plant growth can help to solve this problem. The main parameters that affect the germination ‎rate of various plant seeds are often related to dormancy [3], seed quality [4], physical and biological factors [5], salt and drought stress [6], and seed pretreatment [7]. ‎

Food supply in the future requires the use of new methods in agriculture [8]. Magnetic field application [9,10], X-ray seed priming [11], and gamma radiation application [12,13] are some of the methods that can be used to enhance the performance of crops. Seed priming is one ‎of the methods that can be used to increase seed germination and plant persistence [14]. Seed priming has several advantages, such as increasing germination with a constant rate, ease of application, and inexpensive [15]. There are several methods for seed priming such as hydro priming, hydrothermal priming, matrix priming, and halo ‎priming [16].

One of the environmental stresses that is very important in terms of ‎affecting plants is salt stress. Salinity stress has a negative effect on the growth of many plant ‎species with osmotic, nutritional, and toxic effects [17]. Halopriming can improve seed germination parameters under salt stress [18].

Halopriming of seeds helps repair damaged DNA to prevent damage to the template used for replication and transcription. Halopriming improves salt tolerance by reducing osmotic stress [19]. Priming provides seeds with stress memory, which can be recruited upon subsequent stress exposure by triggering the activation of genes, antioxidant compounds, and transcription factors that increase plant tolerance to salt stress [20].

Different materials can be used for seed priming, including water, phytohormones ‎such as gibberellic acid and salicylic acid, and various salt solutions [21]. Recently, cold plasma technology has received special attention in agriculture, ‎nutrition, biomedicine, environment, and water treatment [22–28]. Recently, the use of cold ‎plasma for seed priming has become popular [24]. In addition to increasing seed ‎germination, plasma-activated water (PAW) can lead to increased plant yield and reduced pathogen ‎growth [29].

Plasma as the fourth state of matter includes positive ions, negative ions, electrons, excited ‎atoms, neutral atoms, and free radicals. Plasma is divided into cold and warm plasma. Cold ‎plasma is a non-equilibrium plasma whose temperature does not exceed 60°C [26,30]. The dielectric ‎barrier discharge (DBD) is one of the most applicable cold atmospheric pressure plasma ‎generation methods. DBD devices are working with green electricity and have simple structures [31]. ‎High voltage is applied to two electrodes that are separated with at least one dielectric. The space ‎between the two electrodes is filled with air or other gases such as oxygen, nitrogen, and argon, ‎which are ionized by the high-voltage electrodes. The existence of a dielectric barrier is very ‎important because it prevents the electric arc formation and allows only small cold discharges. As ‎a result, high energy species (electrons) as well as active species of oxygen and nitrogen and ‎ultraviolet light are produced [32–34].‎

PAW has a direct effect on the surface coating of the seed and may even affect the inner cells of the seed [35]. Seed priming with PAW can affect the metabolic ‎processes involved in plant growth by creating changes in the seed’s surface coating and the ‎penetration of the free radicals into the seed [36,37].

The increase in the percentage of seed germination and plant growth after seed priming and plant ‎irrigation with PAW has been reported in various researches [24,38]. It has been reported that the ‎percentage of Tartary buckwheat seed germination primed with PAW containing reactive oxygen ‎species, increases compared to the control [39]. Junior et al. [40] reported that seeds of the bean ‎‎(*Erythrina velutina*), which had been ‎primed with activated water, demonstrated a greater rate of ‎germination and had a higher vigor ‎index compared to the control. Guragain et al. ‎‎[24] have ‎shown that the germination of soybean seeds primed with PAW has increased compared to the control. The effects of PAW on the rate and percentage of cucumber seed germination ‎and the initial growth of stems and roots were studied, and the results showed that PAW ‎generally increases the germination of cucumber seed [41]. In a research, the effect of PAW on the ‎germination and growth of wheat yield has been investigated and it has been reported that the ‎use of PAW can improve the growth characteristics of wheat, especially in soils that are lacking ‎in nutrients [8]. Song et al. [29] reported that priming with PAW can increase seed vigor and seedling ‎establishment ‎by reducing the adverse effects of environmental stressors such as drought, salinity and ‎pathogen infection. Wheat is one of the plants that has a large cultivation area all over the world and about 700 ‎million tons of this crop are harvested annually for various purposes [42]. The ‎aim of this study was investigation the effects of seed halopriming with PAW ‎using response surface methodology on wheat germination parameters and seedling development.‎

## Materials and methods

### Design and fabrication of DBD plasma device

**Fig 1:**
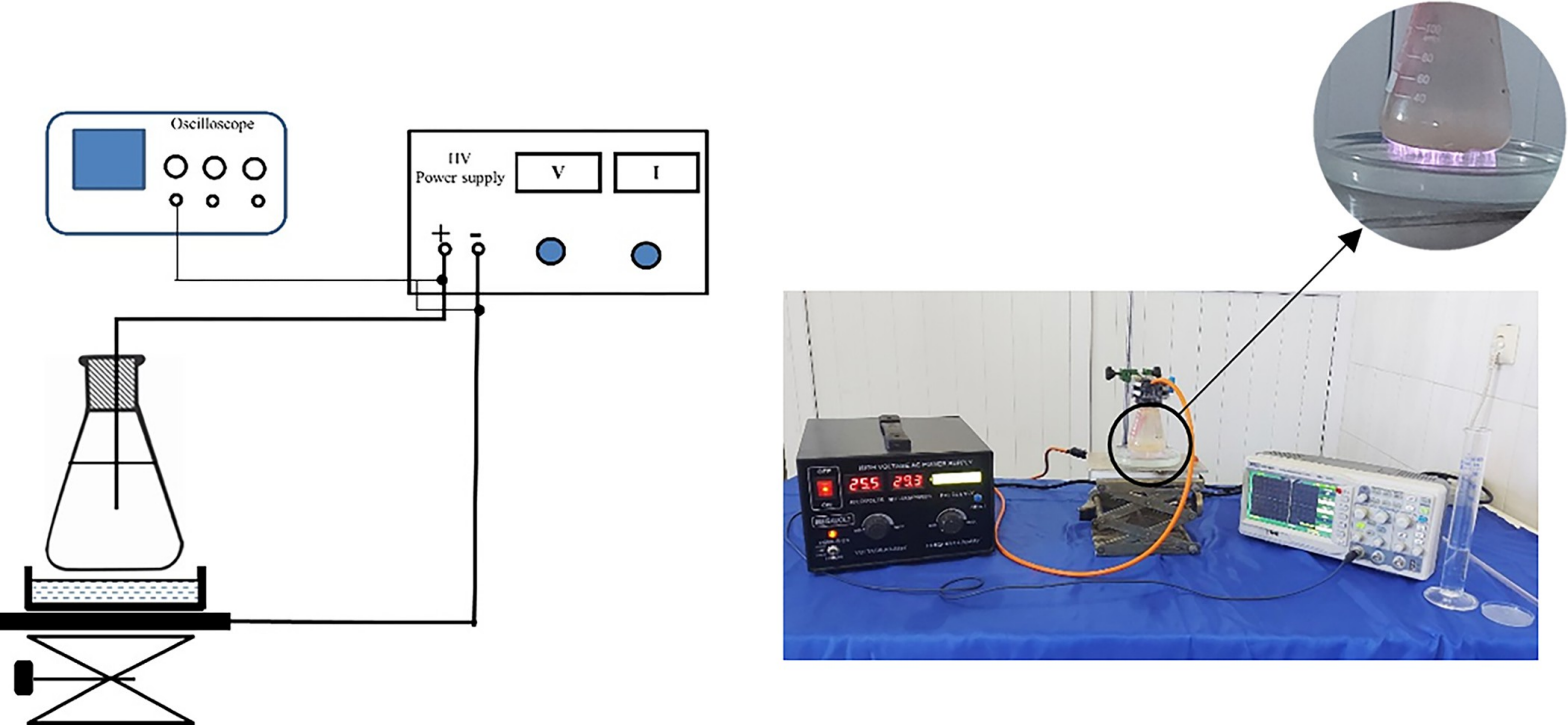
Schematic drawing and picture of DBD plasma activated water generation setup.

A Dielectric Barrier Discharge (DBD) ‎device was designed and constructed to produce PAW in the plasma research laboratory ‎of Tabriz Islamic Azad University. The schematic of the DBD apparatus is shown in Fig 1. One of the electrodes (10 × 20 cm, aluminum) was put under the petri dish and the other (copper wire) was placed inside the Erlenmeyer flask. Erlenmeyer flask and petri dish were filled with water. The applied voltage to the electrodes was 24 kV, with a frequency of 20 ‎kHz. The gap between the upper electrode and the PAW is almost 4 ‎mm. In this research, atmospheric air has been used as a gas in the plasma active medium. The activated plasma water is produced in a petri dish. The activation level of water by plasma has been reported as min/mL.

### Preparation of seeds

The seeds of wheat (*Triticum aestivum* L.) were disinfected for 30 seconds in 70% ethanol and then for 50 seconds in 10% sodium hypochlorite solution and then washed three times with distilled water. Ethanol and sodium hypochlorite were purchased from Jahan Alcohol Teb Arak Co., Iran. Wheat seeds prepared from East Azerbaijan Agricultural and Natural Resources Research Center. All experiments were done in the summer of 2023.

### Cultivation under laboratory conditions

To study the effects of the halopriming with PAW an experiment was carried out in the plasma research laboratory of Islamic Azad University, Tabriz branch, using response surface methodology. The affected factors on wheat germination parameters were PAW at three levels (0, 0.2, and 0.4 min/mL), priming time at three levels (1, 3, and 5 h), and salinity of priming solution at three levels (0, 25, and 50 mmol/L NaCl).

In all experiments (priming and cultivation), distilled water was used for control treatments and PAW was applied for others. The salinity of water samples was made using NaCl (with concentrations of 0, 25 and 50 mmol/L). The petri dishes with a diameter of 15 cm were washed and sterilized in an autoclave at a temperature of 120°C, for 20 minutes. Forty sterilized seeds were primed in 10 mL of water samples for 1, 3, and 5 hours (40 seeds with an average weight of 2 g / 10 mL water), for 17 treatments shown in Table 1. Then the seeds were washed using distilled water and air-dried at ambient temperature. Throughout the seeds soaking, they were regularly aerated using an aquarium pump. Twenty five seeds of every experimental group were placed on a filter paper moistened with 5 mL of water samples and cultivated for 6 days. Subsequently, the dishes were placed within a germination chamber and maintained at 22°C with 60% humidity. Throughout this, the filter papers consistently stayed moist and the number of germinated‎ seeds were counted every day at a designated time. The germination was symbolized by root growth reaching around 2 mm. At the end of cultivation, seedling length, fresh and dry weight of seedlings were measured. The amount of water uptake by seeds was obtained from the difference between the fresh and dry weight of seeds. The following relationships were employed to calculate the germination percentage (PG) [8], mean germination time (MGT) ‎[35], and seed vigor indexes (SVI A and SVI B) [8]‎:

PG=(Ni/N)×100 (1)

$$MGT=\frac{\sum \left(D\times ni\right)}{\sum N}$$ (2)

SVIA=(PG×FW)/100 (3)

SVIB=(PG×DW)/100 (4)

**Table 1:** Selected factors and levels.

Parameter			Levels			
			-1	0		+1
*A*: [PAW]_0_ (min/ mL)			0	0.2		0.4
*B*: Priming Time (min)			60	180		300
*C*: Salinity (mmol/L)			0	25		50
*Run*	Factor1	Factor2			Factor3	
	A: PAW (min/mL)	B: Priming Time (min)			C: Salinity (mmol/L)	
*1*	0.2	180			25	
*2*	0.4	180			0	
*3*	0	60			25	
*4*	0.2	60			50	
*5*	0	180			50	
*6*	0.4	300			25	
*7*	0	300			25	
*8*	0.4	180			50	
*9*	0.2	180			25	
*10*	0.4	60			25	
*11*	0.2	180			25	
*12*	0.2	300			50	
*13*	0.2	180			25	
*14*	0	180			0	
*15*	0.2	180			25	
*16*	0.2	300			0	
*17*	0.2	60			0	

Where Ni and ni are the number of germinated seeds till i^th^day and in i^th^ day, respectively. N is total number of seeds, D is the number of days, ∑*N* is the total number of germinated seeds, DW is the dry weight of the seedling in mg, and FW is the fresh weight of the seedling in mg.‎ Response surface methodology ‎‎(RSM) based on Box Behnken design (BBD) was used for modeling and studying the effect of operational parameters. To model the process, important operational parameters such as PAW (min/mL), priming time (min), and salinity (mmol/L), according to Table 1 and eight responses, germination percentage (%), fresh weight (mg/seedling), dry weight (mg/seedling), total length (cm/seedling), mean germination time (day), vigor index A, vigor index B and water uptake (g/20 seeds) were selected. Analysis of Variance, statistical regression, and response surface analysis were used to find the function between the variables and the responses, and the optimal conditions of the process were determined and confirmed.

In this research, the Design-Expert 7.0.0 software is used to design of experiments. Using selected factors, the software provides 17 experiments (Table 1). All experiments were done in three repetitions and the averages were inserted into the software. The processing of data gives ANOVA tables that provide statistical analysis.

## Results and discussion

Chemical analysis of PAW is shown in Table 2. As shown, the concentration of nitrate ions, TDS, and H_2_O_2_ increased with an increase in plasma activation time, while the pH of the solution decreased. Also, the concentration of ammonium ions in the solution was not changed by increasing of the plasma activation time.

**Table 2:** Chemical analysis of plasma activated water.

Sample		NO_3_^-^ (mg/L)	NH_4_^+^ (mg/L)	TDS (mg/L)	pH	H_2_O_2_ (mg/L)
**Distilled water**	5.298		<0.1	268	7.02	0.0
**PAW 0.1 min/mL**	183.57		<0.1	488	7.36	2.3
**PAW 0.2 min/mL**	302.87		<0.1	562	4.32	4.6
**PAW 0.3 min/mL**	422.2		<0.1	520	3.28	7.7
**PAW 0.4 min/mL**	447.57		<0.1	566	2.42	10
**Analytical Methods**	UV-Spectrophotometric Screening Method, 4500-NO_3_		ASTM D1426-15 A-Direct Nesselerization	Dried at 180°C, 2540-C	pH meter, 4500-H^+^	Colorimetric Method

### Germination percentage

The analysis of variance for germination percentage is given in Table 3.

**Table 3:** ANOVA for germination percentage.

Source	Sum of Squares	Df	Mean Square	F Value	*P*-value Prob > F
**Model**	26443.53	9	2938.17	26.37	0.0001
**A-PAW**	11250	1	11250	100.96	< 0.0001
**B-Time**	0	1	0	0	1
**C- Salinity**	1250	1	1250	11.22	0.0123
**AB**	100	1	100	0.9	0.375
**AC**	100	1	100	0.9	0.375
**BC**	400	1	400	3.59	0.1
**A^2**	3541.05	1	3541.05	31.78	0.0008
**B^2**	4867.37	1	4867.37	43.68	0.0003
**C^2**	3541.05	1	3541.05	31.78	0.0008
**Residual**	780	7	111.43		
**Lack of Fit**	700	3	233.33	11.67	0.019
**Pure Error**	80	4	20		
**Cor Total**	27223.53	16			
**Std. Dev.**	10.56	R-Squared	0.9713		
**Mean**	54.71	Adj R-Squared	0.9345		
**C.V. %**	19.3	Pred R-Squared	0.584		
**PRESS**	11325	Adeq Precision	13.031		

The $R_{Pred}^{2}$ of 0.5840 was not as close to the $R_{Adj}^{2}$ of 0.9345 as one might normally expect. This may indicate a large block effect or a possible problem in the model and/or data. The F-value of 26.37 for the model implies that the model is significant and there is only a 0.01% chance ‎that‏ ‏it occurs due to noise.‎‏Values of P less than 0.0500 indicate model terms are significant. In this case, A, C, A2, B2, and C2 are significant model terms.

A proper mathematical model between independent variables (PAW levels, priming time, and salinity levels) and germination percentage was obtained. The obtained equation is shown as follows:

$$Germination\;Percentage=+98.00‐37.50*A+0.000*B‐12.50*C+5.00*A*B+5.00*A*C+10.00*B*C‐29.00*A^{2}‐34.00*B^{2}‐29.00*C^{2}$$ (5)

**Fig 2:**
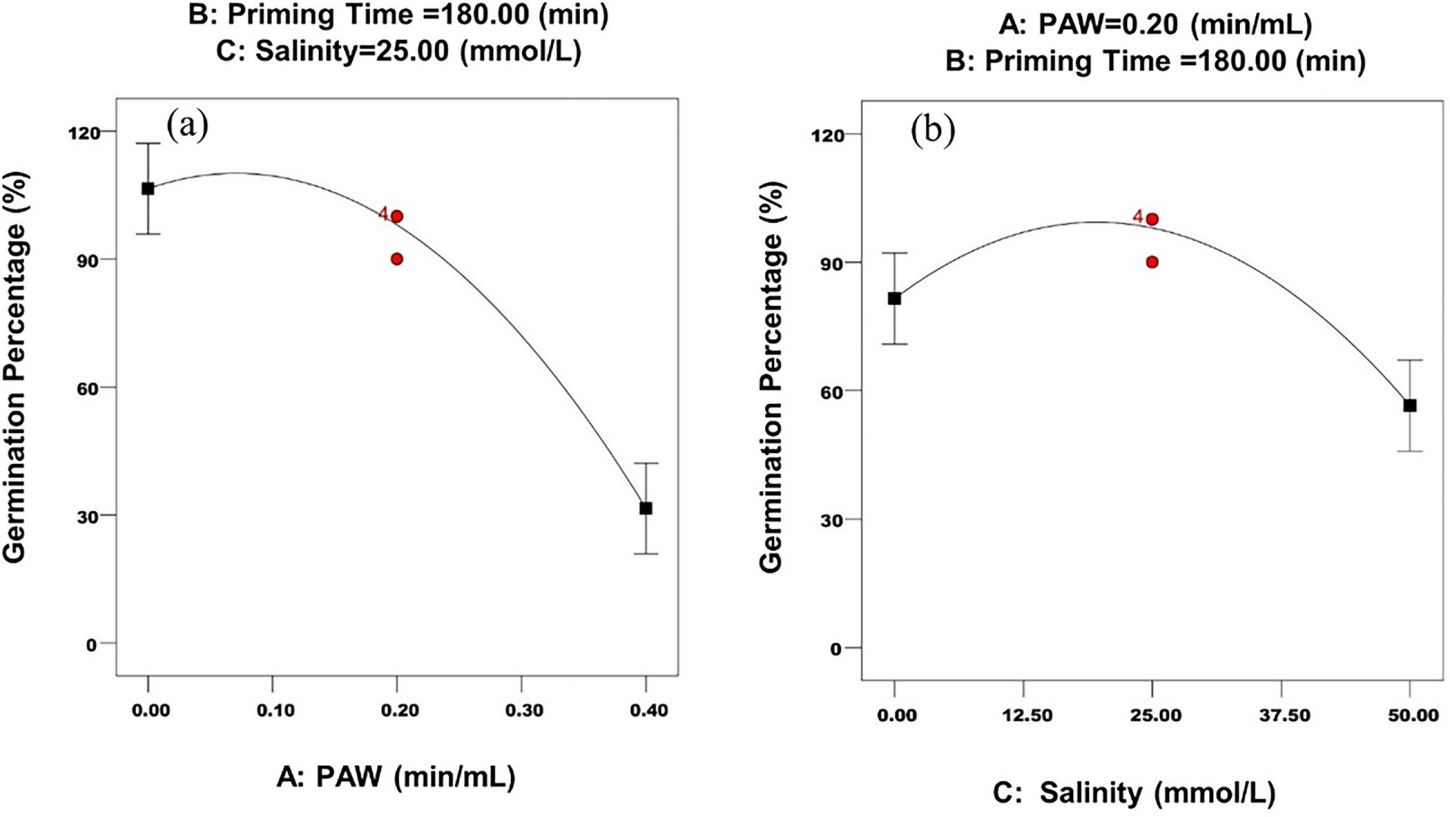
The main effects of a) PAW and b) salinity on germination percentage.

The main effect of PAW on germination percentage is shown in Fig 2A. As shown in Fig 2A, the germination percentage increases with the elevation of PAW levels, up to 0.13 min/mL (Table 4), and then decreases with increasing PAW levels. Kucerova et al. [8] have examined the effects of five PAW levels (0, 0.5, 1, 1.5, and 2 min/mL) on wheat germination and reported that the highest germination percentage of seeds was observed at the 1 min/mL level and germination percentage decreased by increasing the PAW levels. In that level of PAW, the concentration of H_2_O_2_ was about 20–22 mg/L and the concentration of NO_3_‾ was about 31 mg/L. However, it is reported that H_2_O_2_, NO_2_^‾,^ and NO_3_‾ concentration changes are different without and in contact with seeds [8]. Degradation of these species are faster in contact with seeds, probably they are metabolized by seeds once they start to germinate [8]. The enhanced effects of PAW on seed germination could be due to the increasing absorption of H_2_O_2_ by the seed during the priming stage or the later phases of seed growth. The effect of the H_2_O_2_ accumulation in early growth stages has been documented for numerous plant species [43,44]. H_2_O_2_ can increase the respiration and metabolic activities of the seed through oxygen production, it can also facilitate the entry of water into the seed and oxidize germination inhibitors [45,46]. The improving effect of PAW on seed growth parameters can also be due to the existence of other species present such as ^•^O_2_^-^ or ^•^OH in it [47,48]. On the other hand, reports are indicating that important nitrogen species such as NO_3_‾ [49–51] and reactive nitrogen species [50,52,53] can stimulate the germination of plants and decrease the duration of seed dormancy.

**Table 4:** Optimal conditions for affecting the germination percentage.

PAW (min/mL)	Time (min)	Salinity (mmol/L)	R1	Desirability
0.13	120.22	10.3	101.282	1

The effect of nitrogen species on the growth parameters of seeds is attributed to the adjustive effects of these species on the amount of plant hormones such as abscisic acid and gibberellic acid [54]. The production and metabolism of reactive species are in dynamic equilibrium [55]. By interfering with the abscisic acid/ gibberellic acid signaling pathways, generated reactive species in PAW may operate as positive signal molecules, reducing the seed latency period and accelerating seed sprouting, resulting in enhanced seed germination [56].

The effect of the PAW on germination also depends on the type of plant species, type of discharge, type of plasma treatment (direct or indirect), type of water (deionized or tap), experimental conditions (in vitro or in vivo), etc. [8]. For example, Lindsay et al. [57] found a non-significant difference in the germination rate of radish, tomato, and marigolds grown in pots with soil and irrigated with the PAW compared to the control, while Zhang et al. [58] observed 50% improvement in germination of lentil seeds irrigated in vitro with the PAW produced by atmospheric pressure He plasma jet.

High PAW levels (more than 0.2 min/mL), lead to a decrease in germination percentage (Fig 2A). Increasing the PAW level leads to produce of high concentrations of active oxygen and nitrogen species. And they have a high negative effect on the potential of the PAW (Table 2). Furthermore, elevated levels of NO_3_^-^ within PAW can potentially exert an adverse effect on the germination percentage of seeds [59]. Furthermore, high amount of acidity in high PAW levels can be harmful for seeds (Table 2). Table 4 shows the optimal conditions that affect the germination percentage.

The main effect of salinity on germination percentage is shown in Fig 2B. As can be seen in Fig 2B, ‎with increasing the salinity level up to 10.3 (Table 4)‎, the germination percentage increases, and with a further increase in the salinity, the germination percentage decreases. Salinity decreases growth parameters, through complex traits that include osmotic stress, ion toxicity, mineral deficits, and physiological and biochemical defects [60]. Rajabi Dehnavi et al. [61], studied the effect of salinity on seed germination and seedling development of sorghum genotypes and concluded that, with salinity increasing to 100 mmol/L NaCl, germination percentage in some genotypes decreased between 5% and 9% but under 150 and 200 mmol/L NaCl, germination percentage was significantly reduced in all sorghum genotypes.

It has been reported that under salt stress, the conversion of starch to simpler sugars increases, with the aim of maintaining more osmotic balance. Also, seeds under salinity stress uptake more sodium and chlorine ions to maintain their osmotic balance, all of which can disrupt germination and reduce seed growth and establishment [62].

### Seedling fresh weight

Table 5 shows the analysis of variance for seedling fresh weight.

**Table 5:** ANOVA for seedling fresh weight.

Source	Sum of Squares	Df	Mean Square	F Value	*P*-value Prob > F
**Model**	1478.19	9	164.24	283.88	< 0.0001
**A-PAW**	28.13	1	28.13	48.61	0.0002
**B-Time**	78.12	1	78.12	135.03	< 0.0001
**C- Salinity**	242	1	242	418.27	< 0.0001
**AB**	30.25	1	30.25	52.28	0.0002
**AC**	16	1	16	27.65	0.0012
**BC**	4	1	4	6.91	0.0339
**A^2**	402.32	1	402.32	695.37	< 0.0001
**B^2**	222.84	1	222.84	385.16	< 0.0001
**C^2**	342.95	1	342.95	592.75	< 0.0001
**Residual**	4.05	7	0.58		
**Lack of Fit**	3.25	3	1.08	5.42	0.0681
**Pure Error**	0.8	4	0.2		
**Cor Total**	1482.24	16			
**Std. Dev.**	0.76	R-Squared	0.9973		
**Mean**	78.53	Adj R-Squared	0.9938		
**C.V. %**	0.97	Pred R-Squared	0.9641		
**PRESS**	53.25	Adeq Precision	44.439		

The results show a high value of coefficient of determination ($R_{Pred}^{2}=0.9641$ and $R_{Adj}^{2}=0.9938$ 0.9938). The high F-value (283.88) and P<0.0500 in Table 4 show the significance of model. As results, the terms A, B, C, AB, AC, BC, A2, B2, C2 are significant. The obtained equation between seedling fresh weight and independent factors is shown as following:

$$Seedling\;fresh\;weight=+90.80‐1.88*A+3.12*B‐5.50*C‐2.75*A*B+2.00*A*+1.00*B*C‐9.77*A^{2}‐7.27*B^{2}‐9.03*C^{2}$$ (6)

**Fig 3:**
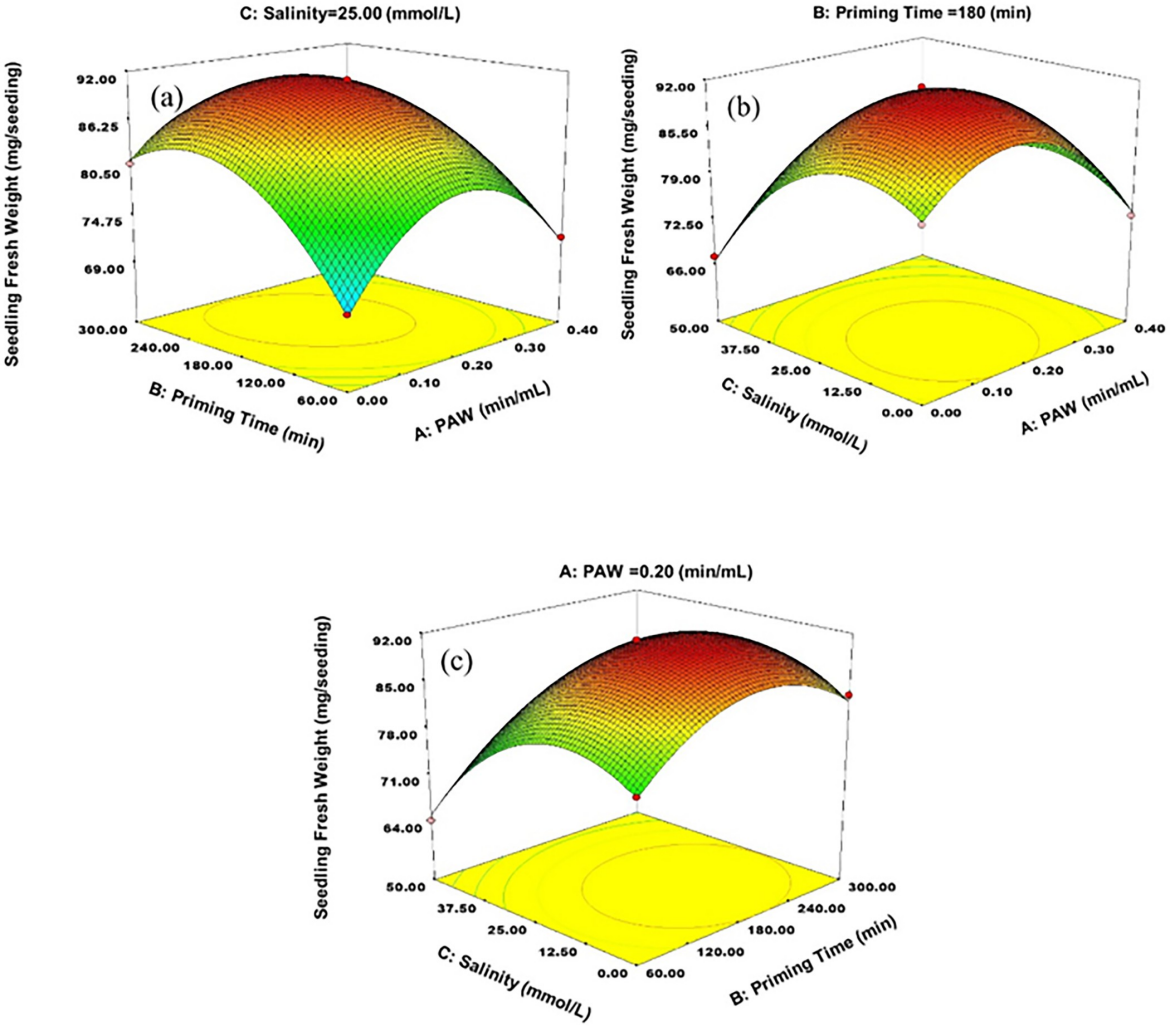
Interaction effects of a) PAW × time, b) PAW × salinity and c) time × salinity on seedling fresh weight.

In order to investigate the interaction effects of the factors on seedling fresh weight, the RSM was used, and 3D plots are shown in Fig 3.

As shown in Fig 3A and 3B, increasing PAW levels could increase the fresh weight of wheat seedlings. This enhancement was evident up to the level of 0.18 min/mL (Table 6) of PAW and then, with increasing PAW level, the fresh weight of the seedling decreased.

**Table 6:** Optimal conditions for fresh weight of the seedling.

PAW (min/mL)	Time (min)	Salinity (mmol/L)	R1	R2
0.18	191.24	15	101.836	91.9561

As the activation time of water increases, the concentration of nitrate, nitrite, ammonium and also the amount of EC (electrical conductivity or salinity) in PAW increases and pH decreases [35]. All these factors have negative effects on growth parameters of wheat seedlings, including fresh weight. In a similar study, the highest fresh weight of wheat seedlings was observed in the PAW treatment equal to 0.5 min/mL, and in other treatments (both lower and higher treatments), the fresh weight of the seedlings showed a significant decrease [8]. According to the results, halopriming (up to 15 mmol/L) could increase fresh weight of seedlings. Seed priming as a pre-germinative treatment might create a moderate stress signal that elicits a “priming memory” to help plants cope with subsequent stress and improve stress tolerance [63]. As shown in Fig 3B and 3C, by increasing the salinity levels to more than 25 mmol/L NaCl, the fresh weight of the seedlings decreased. As shown in Fig 3B, the fresh weight of the seedling at the 0.18 min/mL level of PAW, in all salinity levels, had the highest value. This means that applying this level of PAW can increase wheat seedlings tolerance to salinity.

As shown in Fig 3A and 3C, the optimal time of priming was in the range of 130 to 270 minutes. According to Fig 3C, with increasing salinity levels, the range of the best time of priming, narrows, in the other hand, with the increase of priming time and salinity, the fresh weight of wheat seedlings decreases. According to Table 6, the highest fresh weight of wheat seedlings was obtained at the 0.18 min/mL and 191.24 minutes. At all levels of priming time, decreasing or increasing the PAW levels from 0.18 min/mL led to ‎a decrease in the fresh weight of seedlings.

### Seedling dry weight

According to the Table 7, the $R_{Pred}^{2}$ of 0.9928 is in reasonable agreement with the $R_{Adj}^{2}$ of 0.9990. The obtained F-value and P > F implies the model is significant (Table 6), and the terms A, B, C, AB, AC, BC, A2, B2, C2 are significant. The equation between seedling dry weight and independent factors is shown as following:

$$Seedling\;dry\;weight=+61.00‐0.25*A+3.63*B‐5.13*C‐0.75*A*B‐0.75*A*C‐0.50*B*C‐6.25*A^{2}‐0.50*B^{2}‐3.50*C^{2}$$ (7)

**Table 7:** ANOVA for seedling dry weight.

Source	Sum of Squares	Df	Mean Square	F Value	*P*-value Prob > F
**Model**	552.22	9	61.36	1718.02	< 0.0001
**A-PAW**	0.5	1	0.5	14	0.0072
**B-Time**	105.12	1	105.12	2943.5	< 0.0001
**C-Salinity**	210.13	1	210.13	5883.5	< 0.0001
**AB**	2.25	1	2.25	63	< 0.0001
**AC**	2.25	1	2.25	63	< 0.0001
**BC**	1	1	1	28	0.0011
**A^2**	164.47	1	164.47	4605.26	< 0.0001
**B^2**	1.05	1	1.05	29.47	0.001
**C^2**	51.58	1	51.58	1444.21	< 0.0001
**Residual**	0.25	7	0.036		
**Lack of Fit**	0.25	3	0.083		
**Pure Error**	0	4	0		
**Cor Total**	552.47	16			
**Std. Dev.**	0.19	R-Squared	0.9995		
**Mean**	56.18	Adj R-Squared	0.999		
**C.V. %**	0.34	Pred R-Squared	0.9928		
**PRESS**	4	Adeq Precision	145.747		

**Fig 4:**
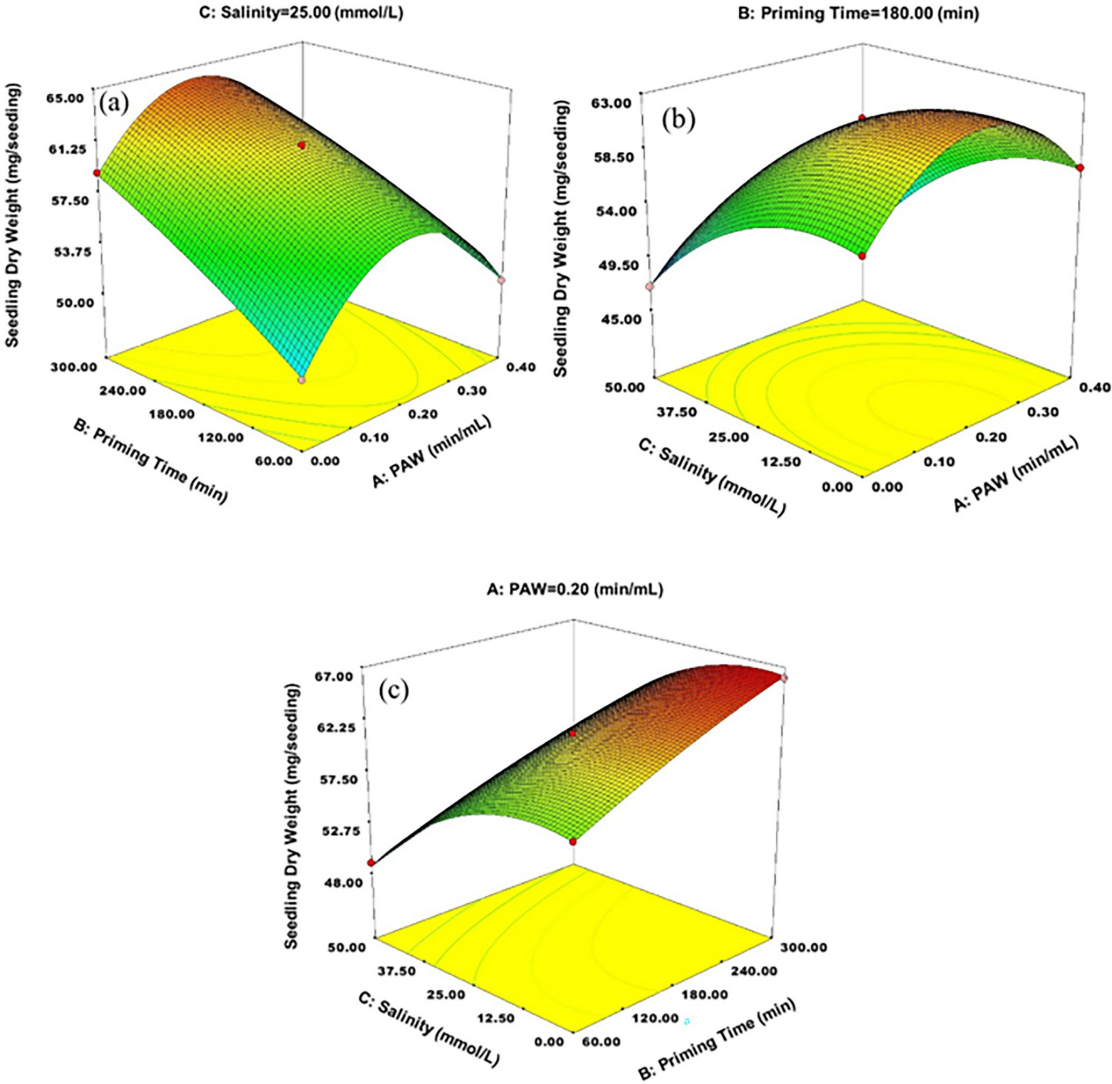
Interaction effects of a) PAW × time, b) PAW × salinity and c) time × salinity on seedling dry weight.

In order to investigate the interaction effects of the factors on seedling dry weight, the RSM was used and 3D plots are shown in Fig 4.

As shown in Fig 4A, in each of the time levels, with the increase of PAW level, the dry weight of the seedling, initially increases and begins to decrease by more increasing PAW level. According to the Table 8, the optimal PAW level for seedling dry weight, is 0.15 min/mL. However, it’s noticeable that as the priming time increases, the optimal PAW limit also increases. So, at 221 minutes, the level of 0.2 min/mL had the most effect on dry weight of seedling. In each of the PAW levels, the dry weight of the seedling increased with the increasing priming time, but its improving effects decreased with increasing the PAW levels. The metabolic process of seedling growth is influenced by reactive nitrogen-oxygen species in PAW, can cause changes on the seed surface and allow radicals to enter the seed, resulting in an improved dry matter of seedlings [24].

**Table 8:** Optimal conditions for seedling dry weight.

PAW (min/mL)	Time (min)	Salinity (mmol/L)	R1	R3	Desirability
0.15	221.2	13.84	100	63.4991	0.939

According to the Fig 4B, increasing the salinity level at all PAW levels, leads to a reduction in the dry weight of the seedling. At the level of 0.2 min/mL, dry weight of seedling was the highest amount compared to the other PAW treatments, in each of the salinity levels. This means that applying this level of PAW can make wheat seedlings more tolerant to salinity. According to Fig 4C, in each of the time levels, increasing salinity level caused to decreasing seedling dry weight and in each of the salinity levels, increasing the priming time caused to increasing seedling dry weight. Robledo et al. [64] studied the effects of halopriming with NaCl (0, 10, 25, and 50 mg/L) and KCl (0, 10, 25, and 50 mg/L) on the germination parameters of bell pepper seeds. The greatest influence on the fresh and dry weight of the seedling was observed when the seeds were primed for 24 hours using 25 mg/L of KCl. The reason was attributed to the early failure of seed reserves due to the premature breakdown of cell wall proteins, and it was reported that the lower effect of halopriming with sodium chloride is related to the toxic effects of sodium ions.

### Mean germination time

According to the Table 9, the $R_{Pred}^{2}$ of 0. 313 was not as close to the $R_{Adj}^{2}$ of 0.7101 as one might normally expect. This may indicate a large block effect or a possible problem with model and/or data. The F-value of 5.36 implies the model is significant. Values of P less than 0.0500 indicate A, A2 terms are significant. The relation between mean germination time and independent factors is obtained as:

$$Mean\;germination\;time=+4.36‐1.97*A+0.57*B‐0.54*C‐0.020*A*B+0.20*A*C+0.68*B*C‐1.54*A^{2}‐0.92*B^{2}‐0.76*C^{2}$$ (8)

**Table 9:** ANOVA for mean germination time.

Source	Sum of Squares	Df	Mean Square	F Value	*P*-value Prob > F
**Model**	55.78	9	6.2	5.36	0.0189
**A-PAW**	31.17	1	31.17	26.93	0.0013
**B-Time**	2.62	1	2.62	2.27	0.176
**C- Salinity**	2.3	1	2.3	1.99	0.2014
**AB**	1.60E-03	1	1.60E-03	1.38E-03	0.9714
**AC**	0.16	1	0.16	0.13	0.7243
**BC**	1.82	1	1.82	1.57	0.2498
**A^2**	10.03	1	10.03	8.67	0.0216
**B^2**	3.57	1	3.57	3.09	0.1223
**C^2**	2.46	1	2.46	2.12	0.1885
**Residual**	8.1	7	1.16		
**Lack of Fit**	3.41	3	1.14	0.97	0.4898
**Pure Error**	4.69	4	1.17		
**Cor Total**	63.88	16			
**Std. Dev.**	1.08	R-Squared	0.8732		
**Mean**	2.84	Adj R-Squared	0.7101		
**C.V. %**	37.87	Pred R-Squared	0. 313		
**PRESS**	61.89	Adeq Precision	6.534		

**Fig 5:**
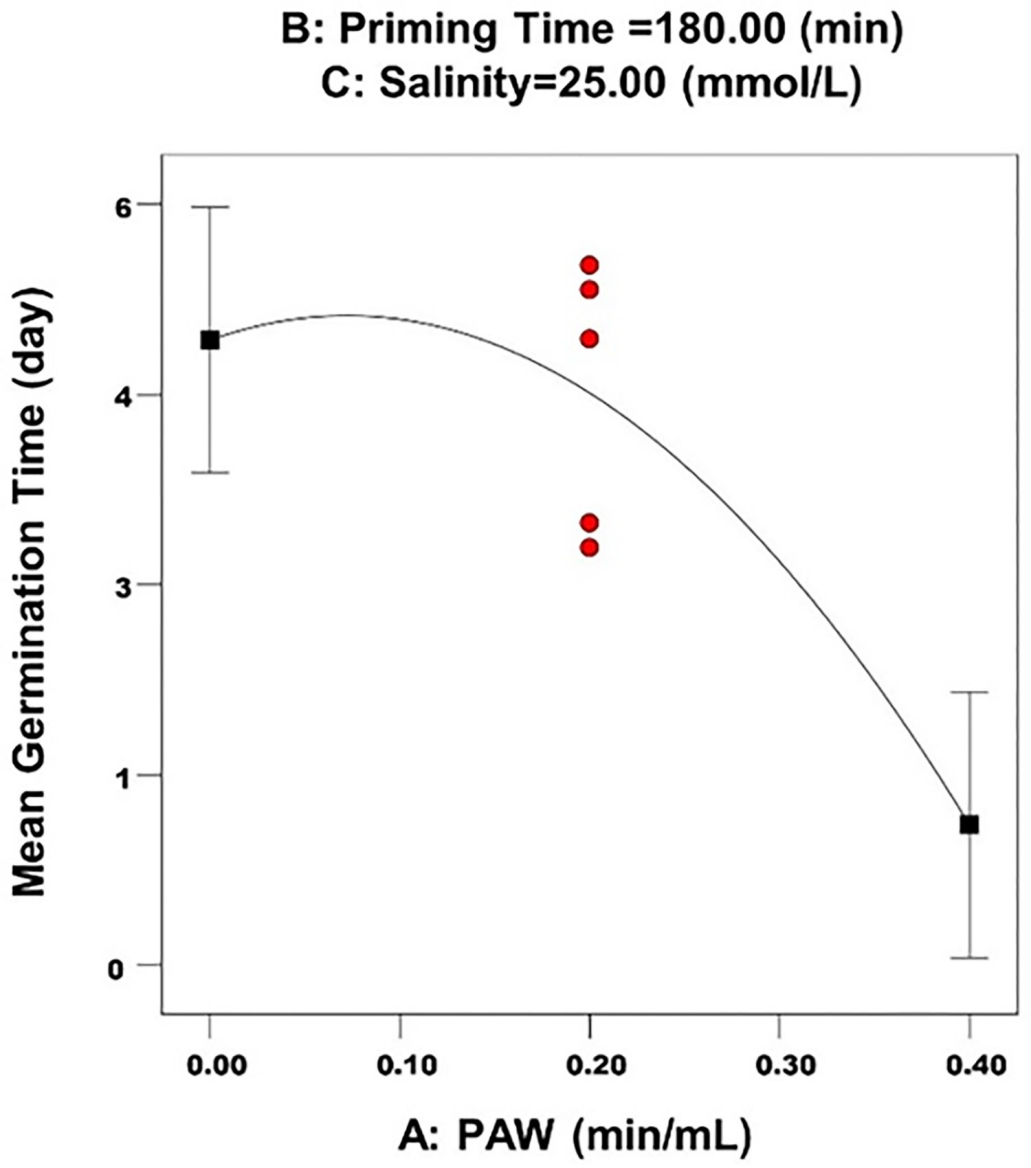
The main effect of PAW on the mean germination time.

Table 10 shows the optimal rates of factors that affect mean germination time. The main effect of synthesis on the mean germination time is shown in Fig 5.

**Table 10:** Optimal conditions for mean germination time.

PAW (min/mL)	Time (min)	Salinity (mmol/L)	R1	R5	Desirability
0.33	152.62	24.36	59.681	2.2914	0.586

As can be seen in Fig 5, increasing PAW levels caused a reduction in mean germination time. Seeds uptake a lot of water during cultivation. PAW has been able to accelerate germination by helping to uptake more water and improving germination interactions. In various studies, the effects of different priming types on reduction of mean germination time, has been reported.

According to research about the comparison of germination speed in tef for untreated, hydroprimed, and gas plasma-activated water (GPAW) primed grain, it was shown that under an optimal germination temperature (32°C), untreated and primed grains showed no significant difference in their germination speed. Favorable conditions (16°C, 20°C) showed slightly faster germination in primed grains compared to the untreated control, but under suboptimal conditions (12°C) GPAW primed grains germinated faster [65].

Guragain et al. [35], investigated effects of plasma-activated water on wheat germination and seedling development, and reported that seeds of wheat irrigated using PAW sprout faster than seeds irrigated with DIW (deionized water). In this research the concentration of NO_3_^-^ were 1.43 and 4.79 mg/L respectively in PAW levels 5 and 10 min/ml and the seeds mean germination time in both levels were significantly less than the control.

The production and metabolism of reactive species are consistently believed to be in dynamic equilibrium [23]. By interfering with the abscisic acid/gibberellic acid signaling pathways, reactive species (RONs) generated in plasma-treated water may operate as positive signal molecules, reducing the seed latency period and accelerating seed sprouting, resulting in an enhanced seed germination [56]. The metabolic process of plant growth is influenced by long-lived reactive nitrogen-oxygen species produced in PAW, which cause changes on the seed surface and allow radicals to enter the seed, resulting in an improved germination [23].

### Vigor index A and B

The analysis of variance for vigor indexes A and B are shown in Tables 11 and 12 respectively. In both of them the P is less than 0.0500 and the obtained F-value implies the models are significant. The results of $R_{Pred}^{2}$, and $R_{Adj}^{2}$ indicate a large block effect or a possible problem with the model (Vigor indexes A and B). The terms A, C, A2, B2, C2 are significant in both vigor indexes. Eqs 9 and 10 show the polynomial relations between response and independent factors in vigor indexes A, and B, respectively:

$$Vigor\;index\;A=+89.60‐27.88*A+1.00*B‐11.63*C+1.00*A*B+5.25*A*C+7.00*B*C‐30.93*A^{2}‐31.67*B^{2}‐29.92*C^{2}$$ (9)

$$Vigor\;index\;B=+58.20‐19.88*A+1.13*B‐8.50*C+0.000*A*B+3.25*A*C+5.25*B*C‐19.85*A^{2}‐18.35*B^{2}‐18.60*C^{2}$$ (10)

**Table 11:** ANOVA for vigor index A.

Source	Sum of Squares	Df	Mean Square	F Value	*P*-value Prob > F
**Model**	21050.49	9	2338.94	19.57	0.0004
**A-PAW**	6216.13	1	6216.13	52.02	0.0002
**B-Time**	8	1	8	0.067	0.8033
**C**-Salinity	1081.13	1	1081.13	9.05	0.0197
**AB**	4	1	4	0.033	0.86
**AC**	110.25	1	110.25	0.92	0.3688
**BC**	196	1	196	1.64	0.2411
**A^2**	4026.76	1	4026.76	33.7	0.0007
**B^2**	4224.44	1	4224.44	35.35	0.0006
**C^2**	3770.55	1	3770.55	31.55	0.0008
**Residual**	836.45	7	119.49		
**Lack of Fit**	363.25	3	121.08	1.02	0.471
**Pure Error**	473.2	4	118.3		
**Cor Total**	21886.94	16			
**Std. Dev.**	10.93	R-Squared	0.9618		
**Mean**	46.06	Adj R-Squared	0.9126		
**C.V. %**	23.73	Pred R-Squared	0.7007		
**PRESS**	6551.38	Adeq Precision	11.343		

**Table 12:** ANOVA for vigor index B.

Source	Sum of Squares	Df	Mean Square	F Value	*P*-value Prob > F
**Model**	8966.69	9	996.3	10.29	0.0028
**A-PAW**	3160.13	1	3160.13	32.65	0.0007
**B-Time**	10.13	1	10.13	0.1	0.7558
**C-Salinity**	578	1	578	5.97	0.0445
**AB**	0	1	0	0	1
**AC**	42.25	1	42.25	0.44	0.53
**BC**	110.25	1	110.25	1.14	0.3213
**A^2**	1659.04	1	1659.04	17.14	0.0043
**B^2**	1417.78	1	1417.78	14.65	0.0065
**C^2**	1456.67	1	1456.67	15.05	0.0061
**Residual**	677.55	7	96.79		
**Lack of Fit**	230.75	3	76.92	0.69	0.6047
**Pure Error**	446.8	4	111.7		
**Cor Total**	9644.24	16			
**Std. Dev.**		9.84	R-Squared	0.9297	
**Mean**		31.47	Adj R-Squared	0.8394	
**C.V. %**		31.26	Pred R-Squared	0.5448	
**PRESS**		4390.13	Adeq Precision	8.425	

**Fig 6:**
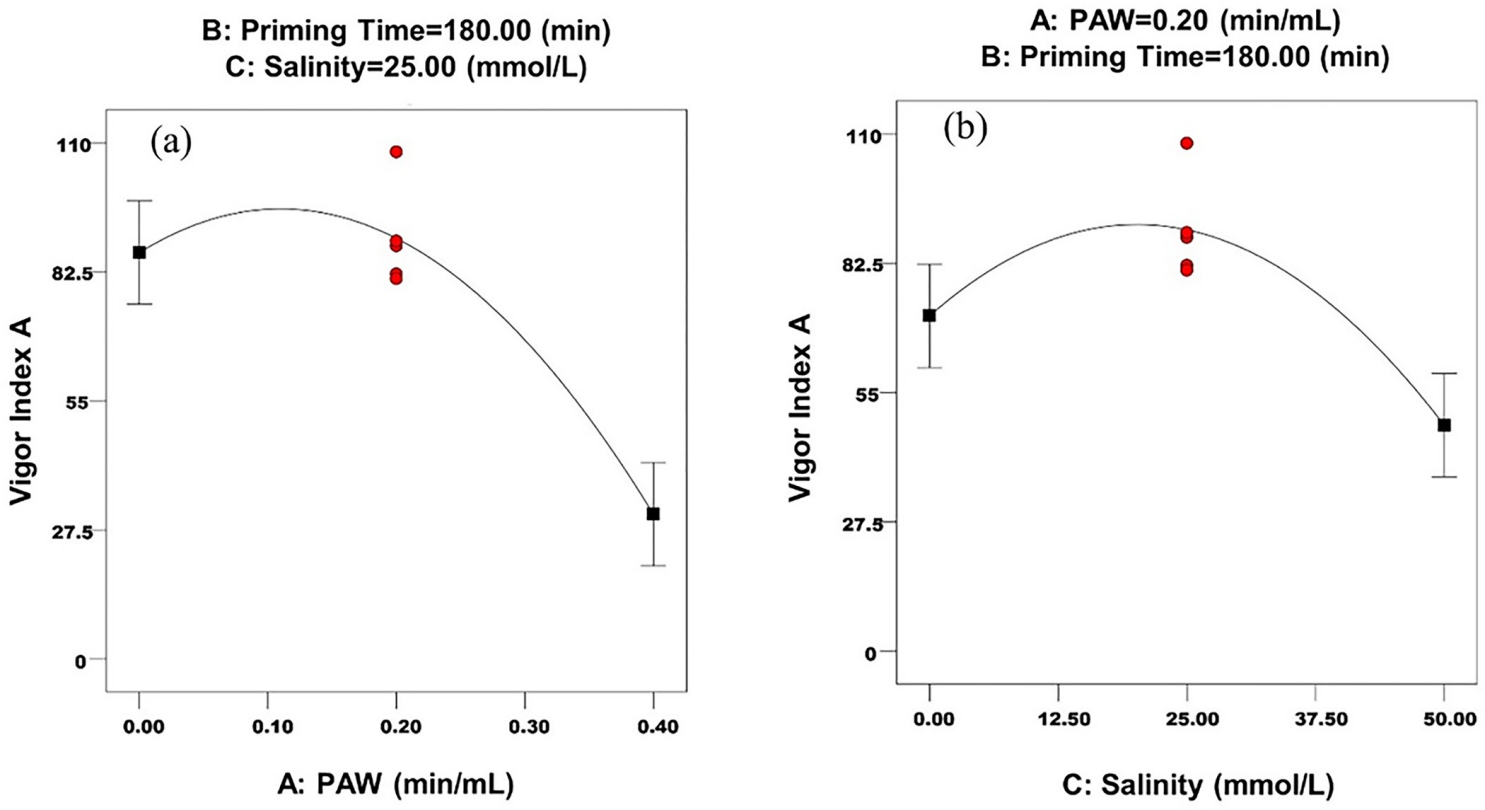
The main effects of a) PAW and b) salinity on the vigor index A.

**Fig 7:**
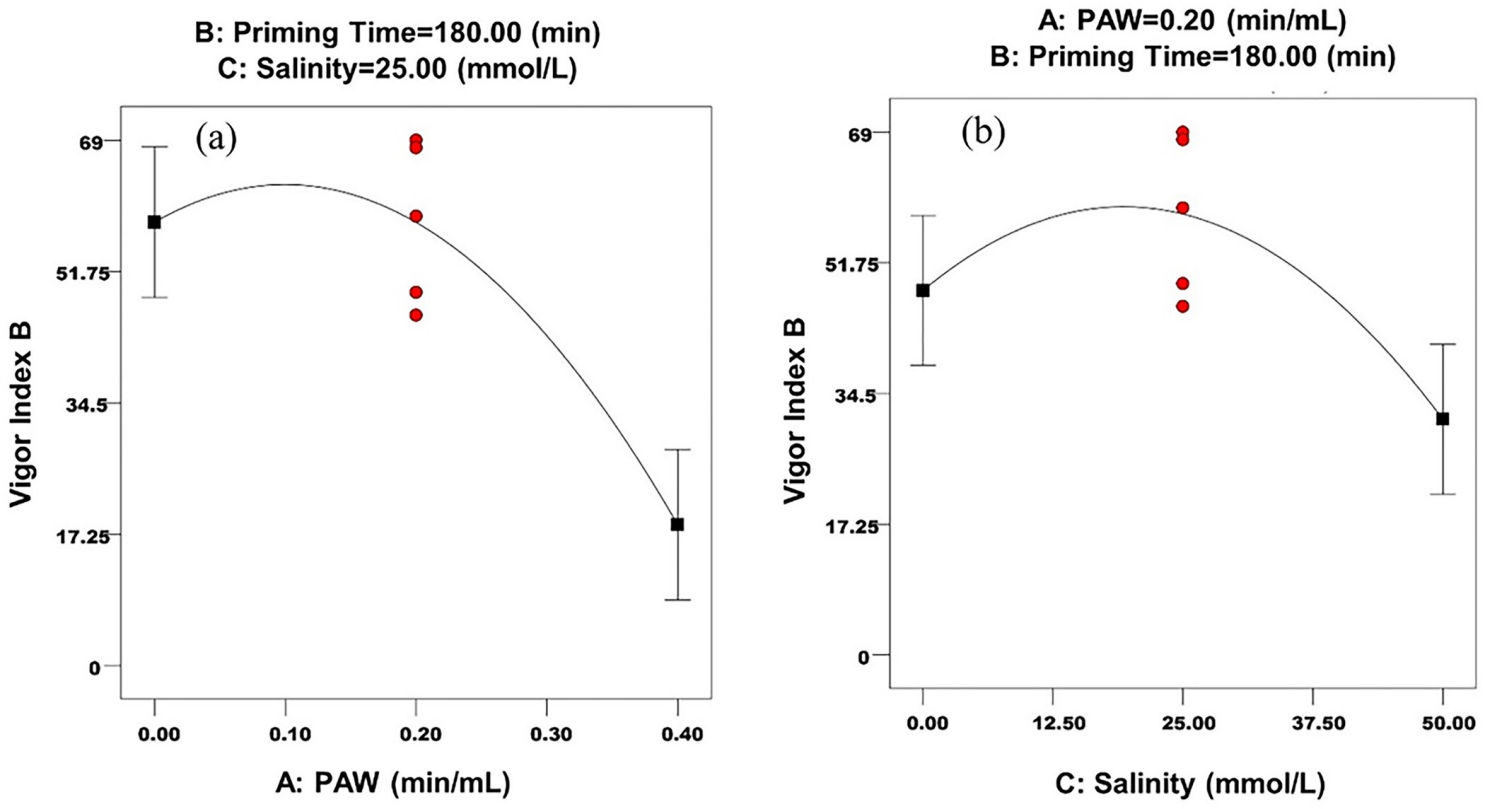
The main effects of a) PAW and b) salinity on vigor index B.

The main effects of PAW and salinity levels on vigor indexes A and B are shown in Figs 6A, 6B, 7A and 7B, respectively.

Optimal rates of factors for vigor indexes A and B are shown in Table 13.

**Table 13:** Optimal conditions for vigor indexes A and B.

	PAW (min/mL)	Time (min)	Salinity (mmol/L)	R1	R2	Desirability
**Vigor index A**	0.11	177.77	19.03	111.2	97.5391	0.95
**Vigor index B**	0.1	178.99	18.11	111.696	64.5647	0.967

As can be seen in Figs 6A and 7A, PAW increased the vigor indexes A and B, which can be attributed to increased water uptake. After a certain level, the increase in PAW levels has caused a decrease in these parameters. The optimum level of PAW for vigor indexes A and B was 0.1 min/min (Table 13). Junior et al. [40], showed that priming chickpea seeds with PAW at voltages up to 49 kilovolts, resulted in an enhancement in the seed’s vigor index. Rasooli et al. [66], evaluated the effect of priming by PAW for 5 minutes on the physiological characteristics of cumin seeds better than the effect of priming for 0 and 10 minutes, because of more cellular protein synthesis in this treatment. It was additionally reported that in the 10-minute treatment, the activity of protein-synthesizing ribosomes was disrupted and the amount of phenol increased in this treatment, which indicates the production of more antioxidants for greater stability of the cell membrane against excess oxygen and nitrogen species in PAW. In a similar study, the effect of five levels of PAW (0, 0.5, 1, 1.5 and 2 min/mL) on the germination parameters of wheat seeds was investigated and it was reported that the highest A and B vigor indexes, were obtained at the level of 0.5 min/mL. It was observed that with decreasing or increasing of PAW levels, the A and B vigor indexes also decreased. These researchers attributed this effect to better seed nutrition in the presence of active oxygen and nitrogen species in PAW and reported that in the early stages of seed growth, the effect of H_2_O_2_ species on growth is greater, while the metabolism of NO_2_^-^ and NO_3_^-^ species, begin in the next stages of seed development [8]. In the current research, the high concentration of these species and also the high acidity of PAW in higher levels have reduced the vigor indexes compared to the level of 0.1 min/mL (Table 2).

PAW acts on the physiological processes of germination and at high plasma exposure there are stress reactions and reduced germination parameters and death of the seed. However, seeds/fruits treatments with plasma, induced large increase in radical scavenging activity (up to 114%) in leaf extracts [67].

As can be seen in Figs 6B and 7B, halopriming increased the A and B vigor indexes of the seed, up to a certain level and then decreased these parameters. The optimal level of salinity for these parameters, was in the range of 18-19mg/L (Table 13). Robledo et al. [64], reported that the greatest effect of priming with NaCl on the germination parameters of sweet pepper was obtained at the level of 25 mg/L of this treatment, and the levels of 0 and 50 mg/L, caused a decrease in seed germination parameters.

## Conclusion

Cold plasma technology is a promising method that is often used in agriculture, ‎nutrition, biomedicine, environment, and water treatment. In this study the germination percentage, fresh and dry weight of seedlings, seedling length, vigor indexes A and B, and water uptake were all enhanced by elevating PAW levels, reaching a maximum of 0.18 min/mL and then there was a decline in these parameters. The mean germination time of seedling decreased as PAW levels increased. The enhancing effects of PAW on seed germination could be due to the increasing absorption of H_2_O_2_ by the seed during the priming stage or the later phases of seed growth. H_2_O_2_ can increase the respiration and metabolic activities of the seed through oxygen production, it can also facilitate the entry of water into the seed and oxidize germination inhibitors. The effect of PAW on seed growth parameters can also be due to the existence of other species present such as ^•^O_2_^-^ or ^•^OH in it. Furthermore, it can be concluded that increasing the concentration of free oxygen and nitrogen species and also the acidity of PAW at higher radiation levels can damage the cell membrane and reduce seed germination and seedling establishment. Also a high amount of acidity in high PAW levels can be harmful to seeds. Therefore, further researches are needed to determine the best PAW level depends on the plant species and the priming time and to determine the biological effects of PAW on seeds and plants.

The improving effect of halopriming on seedling length was greater than other parameters. In most cases, increasing the salinity level often up to 20 mmol/L NaCl increased the germination parameter and then salinity had inhibiting effect on these parameters. The fresh and dry weight of the seedlings respectively at the 0.18 and 0.2 min/mL levels of PAW, in all salinity levels, had the highest value. This means applying these levels of PAW can reduce salinity effects on these parameters. The optimum range of PAW, salinity and priming time for most investigated parameters was between 0.1–0.18 min/mL, 10–20 mmol/L NaCl and 120–220 minutes, respectively.

## Supporting information

S1 Fig The main effects of a) PAW and b) salinity on seedling length.
(DOCX)

S2 Fig Main effects of a) PAW and b) priming time on water uptake.
(DOCX)

S1 Table ANOVA for seedling length. (DOCX)

S2 Table Optimal rates of factors for seedling length. (DOCX)

S3 Table ANOVA for water uptake. (DOCX)

S4 Table Optimal rates of factors for water uptake. (DOCX)
