# Supplementary material for: Effects of plasma-activated water on germination ‎and initial seedling growth of wheat
Source: PLoS One. 2025 Jan 24;20(1):e0312008. doi: 10.1371/journal.pone.0312008 (PMC11760015; doi:10.1371/journal.pone.0312008)
Supplement: S1 Fig — The main effects of a) PAW and b) salinity on seedling length. (DOCX) [file pone.0312008.s001.docx]

The main effects of PAW and salinity on seedlings length have been shown in Figs S1a and S1b.


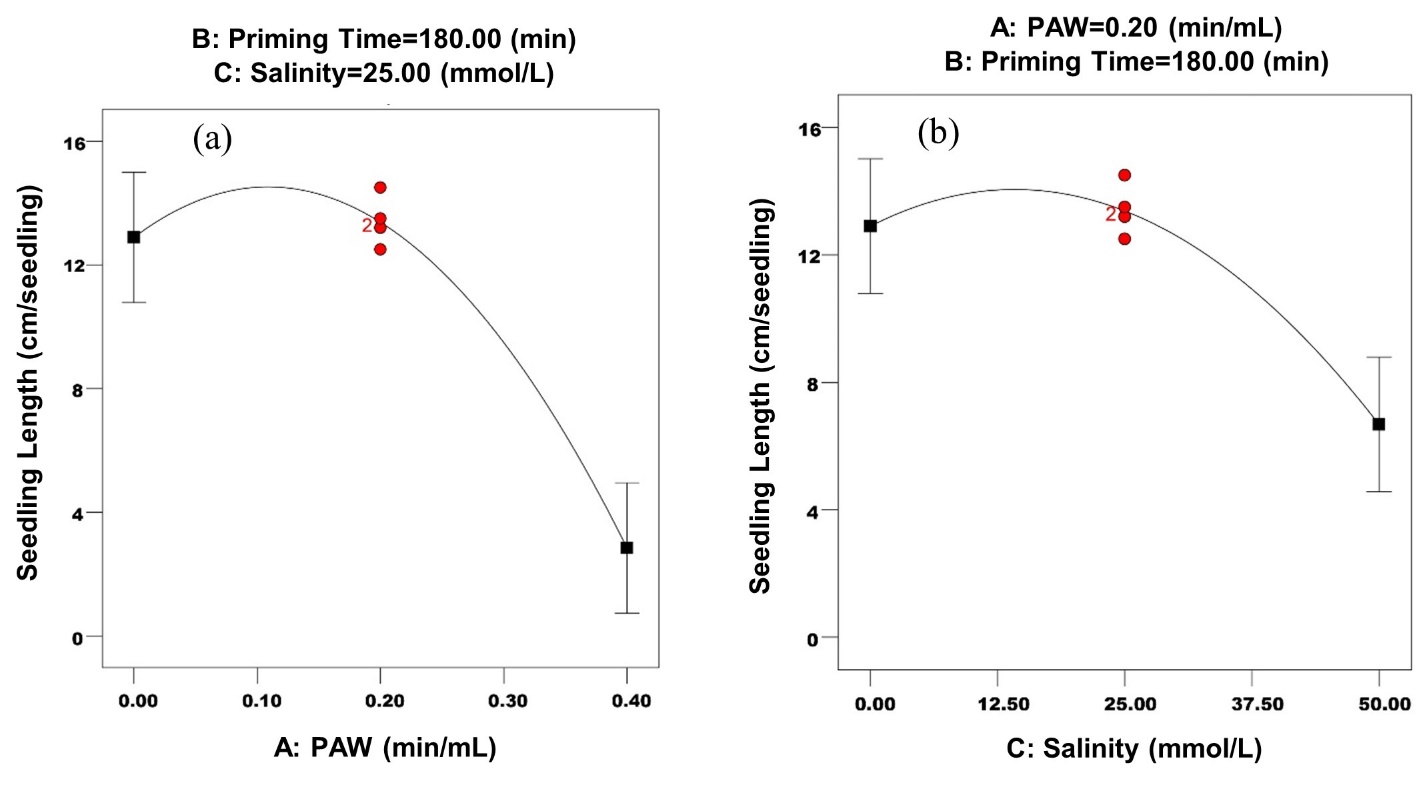


**S1 Fig.** The main effects of a) PAW and b) salinity on seedling length.

According to S1a Fig, PAW up to the level of 0.14 min/ml increased the seedling length, but after that it decreased this parameter.

Guo et al. [1] investigated the effect of PAW with voltages of 0, 9, 11, 13, 15 and 17 kV on seed germination parameters of wheat and concluded that the most positive effect on germination percentage, seed vigor index and seedling length were obtained by 11 kV treatment. Plasma treatment can improve the plant's physiological metabolism by improving enzymes activity and photosynthetic pigments, cause a decrease in seed bacteria growth, change the coating structure and increase the permeability of the coating resulting in stimulated germination and seedling growth [2]. It has been reported that after priming seeds with PAW, the amount of soluble proteins, amylase enzyme and water uptake by seeds increases [1].

Billah et al., [3] reported that the DBD air plasma was involved in the enhancement of nitrogen complex in the seed coat of black gram which upregulated the protein through nitrogen conversion that was ultimately responsible for the increased seed germination and seedling growth of black gram. Reactive oxygen species (ROS) have both positive and negative effects on seed germination and plant growth depending on their doses. The excess of these species may cause oxidative damage which may lead to membrane damage, cell death and subsequently degrade germination rate, growth and production yield. Rasooli et al. [4] investigated the effect of priming by PAW for 0, 5 and 10 minutes on the germination parameters and physiological characteristics of cumin seeds and concluded that the best results were obtained in the priming treatment for 5 min. These researchers announced the destructive effect of long-term seed priming was due to the negative effects of nitrogen and oxygen species in PAW on protein synthesis.

According to Fig S1b, salinity up to the level of 21.9 mg/L was able to increase the length of the seedling, but after that it decreased this parameter. Halopriming has been described as a treatment for pre-sowing in an osmotic solution that permits the seed to absorb water to go to the first stage of germination but prevents radical production via the seed coat. Primed seeds perform better in a wider range of temperatures and are less sensitive to oxygen deprivation than unprimed ones. On the molecular basis, a number of genetic, biological and biochemical processes, including DNA replication and protein synthesis, are implicated in the beneficial influence of priming [5].

However, the rate of increase in seed germination and initial establishment depends on the plant species, as well as the type of saline solution and the dose used. The Negative effects of high salt content on plants are caused by osmotic water retention and strong ionic influences on the protoplasm. Water is held in salt solutions osmotically, therefore, the concentration of increasing salt water is increasingly less accessible for plants. Salinity causes to poor germination and seedling production [5].
