# Supplementary material for: Effects of plasma-activated water on germination ‎and initial seedling growth of wheat
Source: PLoS One. 2025 Jan 24;20(1):e0312008. doi: 10.1371/journal.pone.0312008 (PMC11760015; doi:10.1371/journal.pone.0312008)
Supplement: S2 Fig — Main effects of a) PAW and b) priming time on water uptake. (DOCX) [file pone.0312008.s002.docx]

The main effects of PAW and priming time on water uptake have been shown in Figs S2a and S2b.


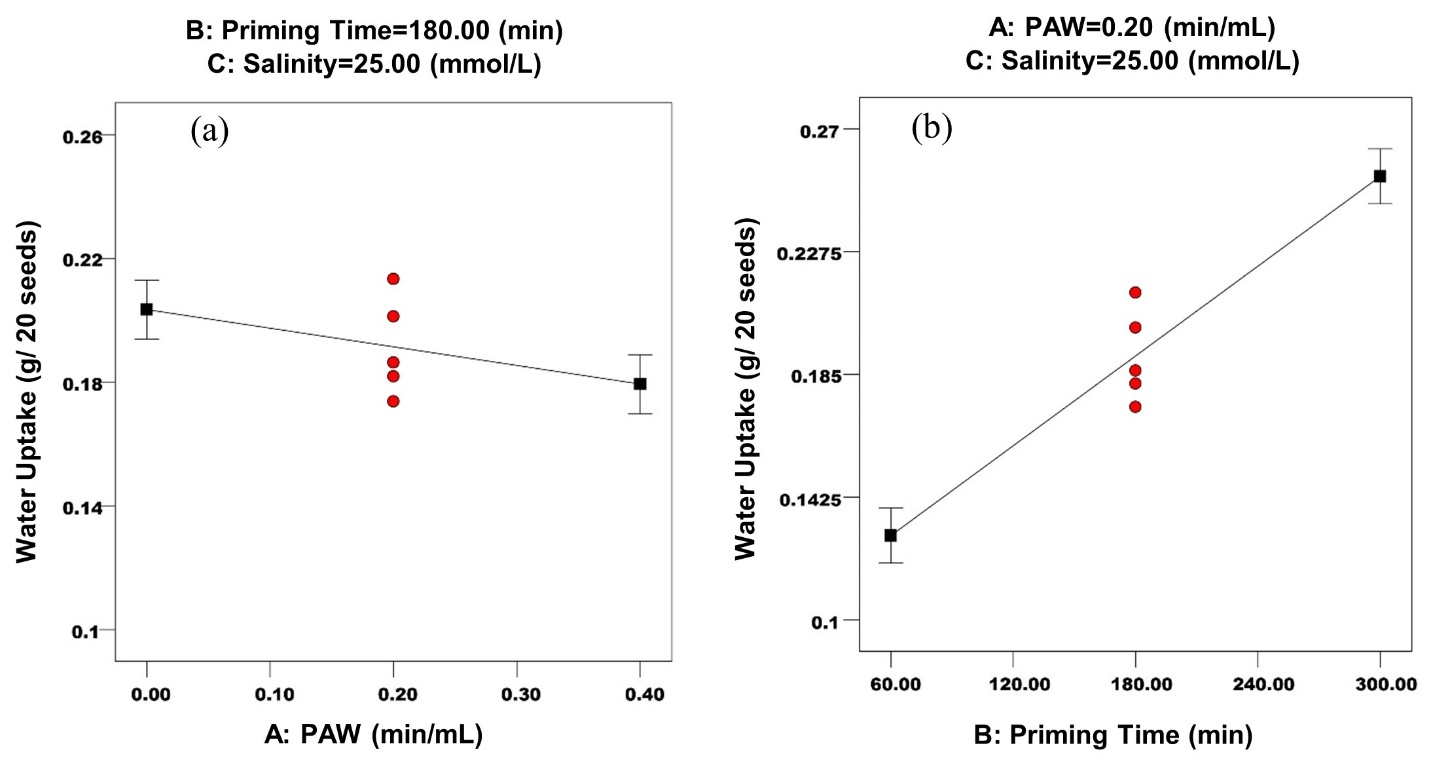


**S2 Fig.** Main effects of a) PAW and b) priming time on water uptake.

The optimum plasma water level for water uptake was 0.27 min/ml (Table S4). Higher levels of PAW caused to decrease in water uptake, probably due to the destructive effects on the cell wall and cell membrane. On the other hand, in this research, salinity had an inhibitory role in water uptake due to the osmotic effect, although the effect of salinity on this parameter, was not significant. As shown in Fig 2Sb, increasing the priming time caused more water uptake by the seeds. Water uptake, especially at the beginning stages of seed growth, is one of the important factors that affects all growth parameters of seeds. Water uptake by seeds, especially in saline or dry conditions, is an important factor on which the future yield of the plant depends. Reduction of germination because of salinity and drought stress results in reductions in water uptake, hormone secretions and enzyme activities [6].
