# Supplementary material for: Effects of plasma-activated water on germination ‎and initial seedling growth of wheat
Source: PLoS One. 2025 Jan 24;20(1):e0312008. doi: 10.1371/journal.pone.0312008 (PMC11760015; doi:10.1371/journal.pone.0312008)
Supplement: S1 Table — (DOCX) [file pone.0312008.s003.docx]

The analysis of variance for seedling length are shown in S1 Table.

S1 Table. ANOVA for seedling length.

.

| Source | Sum of  Squares | Df | Mean  Square | F  Value | p-value  Prob > F |
| --- | --- | --- | --- | --- | --- |
| Model | 523.48 | 9 | 58.16 | 13.21 | 0.0013 |
| A-PAW | 202 | 1 | 202 | 45.88 | 0.0003 |
| B-Time | 5.28 | 1 | 5.28 | 1.2 | 0.3097 |
| C- Salinity | 77.5 | 1 | 77.5 | 17.6 | 0.0041 |
| AB | 0.2 | 1 | 0.2 | 0.046 | 0.8363 |
| AC | 8.7 | 1 | 8.7 | 1.98 | 0.2026 |
| BC | 9.61 | 1 | 9.61 | 2.18 | 0.1831 |
| A^2 | 128.06 | 1 | 128.06 | 29.09 | 0.001 |
| B^2 | 18.39 | 1 | 18.39 | 4.18 | 0.0803 |
| C^2 | 54.27 | 1 | 54.27 | 12.32 | 0.0099 |
| Residual | 30.82 | 7 | 4.4 |  |  |
| Lack of Fit | 28.71 | 3 | 9.57 | 18.16 | 0.0086 |
| Pure Error | 2.11 | 4 | 0.53 |  |  |
| Cor Total | 554.3 | 16 |  |  |  |
| Std. Dev. | 2.1 | R-Squared | 0.9444 |  |  |
| Mean | 8.11 | Adj R-Squared | 0.8729 |  |  |
| C.V. % | 25.87 | Pred R-Squared | 0.4653 |  |  |
| PRESS | 462.69 | Adeq Precision | 10.113 |  |  |

The $R_{Pred}^{2}$ of 0.4653 was not as close to the $R_{Adj}^{2}$ of 0.8729 as one might normally expect. This may indicate a large block effect or a possible problem with the model and/or data. The Model F-value of 13.21 and P > F implies the model is significant. In this case A, C, A2, C2 are significant model terms. The obtained equation between seedling length and independent factors is shown as follows:

Seedling length =+13.38-5.02*A+0.81*B-3.11*C-0.23*A*B+1.48*A*C+1.55*B*C
 -5.52*A2-2.09*B2-3.59*C2
