# Supplementary material for: Effects of plasma-activated water on germination ‎and initial seedling growth of wheat
Source: PLoS One. 2025 Jan 24;20(1):e0312008. doi: 10.1371/journal.pone.0312008 (PMC11760015; doi:10.1371/journal.pone.0312008)
Supplement: S2 Table — (DOCX) [file pone.0312008.s004.docx]

Table S2 shows the optimal rates of factors that affect seedling length.

**S2 Table**. Optimal rates of factors for seedling length.

| PAW (min/mL) | Time (min) | Salinity (mmol/L) | R1 | R4 | Desirability |
| --- | --- | --- | --- | --- | --- |
| 0.14 | 182.15 | 21.9 | 107.978 | 14.7984 | 1 |
