# Supplementary material for: Effects of plasma-activated water on germination ‎and initial seedling growth of wheat
Source: PLoS One. 2025 Jan 24;20(1):e0312008. doi: 10.1371/journal.pone.0312008 (PMC11760015; doi:10.1371/journal.pone.0312008)
Supplement: S3 Table — (DOCX) [file pone.0312008.s005.docx]

Table S3 shows the analysis of variance for water uptake.

**S3 Table.** ANOVA for water uptake.

| Source | Sum of  Squares | Df | Mean  Square | F  Value | p-value  Prob > F |
| --- | --- | --- | --- | --- | --- |
| Model | 0.032 | 3 | 0.011 | 50.69 | < 0.0001 |
| A-PAW | 1.16E-03 | 1 | 1.16E-03 | 5.49 | 0.0357 |
| B-Time | 0.031 | 1 | 0.031 | 146.49 | < 0.0001 |
| C- Salinity | 2.15E-05 | 1 | 2.15E-05 | 0.1 | 0.7551 |
| Residual | 2.75E-03 | 13 | 2.11E-04 |  |  |
| Lack of Fit | 1.74E-03 | 9 | 1.93E-04 | 0.77 | 0.6606 |
| Pure Error | 1.01E-03 | 4 | 2.52E-04 |  |  |
| Cor Total | 0.035 | 16 |  |  |  |
| Std. Dev. | 0.015 | R-Squared | 0.9212 |  |  |
| Mean | 0.19 | Adj R-Squared | 0.9031 |  |  |
| C.V. % | 7.6 | Pred R-Squared | 0.863 |  |  |
| PRESS | 4.78E-03 | Adeq Precision | 21.058 |  |  |

The $R_{Pred}^{2}$ of 0.8630 is in reasonable agreement with the $R_{Adj}^{2}$ of 0.9031. The Model F-value of 50.69 implies the model is significant. Values of Prob > F less than 0.0500 indicate model terms are significant. In this case A, B are significant model terms. The obtained equation between water uptake and independent factors is shown as following:

Water uptake=+0.19-0.012*A+0.062*B-1.637E-003*C
