# Supplementary material for: Effects of plasma-activated water on germination ‎and initial seedling growth of wheat
Source: PLoS One. 2025 Jan 24;20(1):e0312008. doi: 10.1371/journal.pone.0312008 (PMC11760015; doi:10.1371/journal.pone.0312008)
Supplement: S4 Table — (DOCX) [file pone.0312008.s006.docx]

Optimal rates of factors for water uptake are shown in Table S 4.

**S4 Table.** Optimal rates of factors for water uptake.

| PAW (min/mL) | Time (min) | Salinity (mmol/L) | R1 | Desirability |
| --- | --- | --- | --- | --- |
| 0.27 | 179.94 | 20.37 | 81.4117 | 0.745 |
